# Analysis of Antigens of *Mycobacterium leprae* by Interaction to Sera IgG, IgM, and IgA Response to Improve Diagnosis of Leprosy

**DOI:** 10.1155/2014/283278

**Published:** 2014-06-29

**Authors:** Avnish Kumar, Om Parkash, Bhawneshwar K. Girdhar

**Affiliations:** ^1^Department of Biotechnology, School of Life Sciences, Dr. Bhim Rao Ambedkar University, Khandari Campus, Agra, Uttar Pradesh 282004, India; ^2^Department of Immunology, National JALMA Institute for Leprosy and Other Mycobacterial Diseases, Tajganj, Agra, Uttar Pradesh 282001, India; ^3^Shanti Manglik Hospital, Fatehabad Road, Agra 282001, India

## Abstract

Till 2010, several countries have declared less than one leprosy patient among population of 10,000 and themselves feeling as eliminated from leprosy cases. However, new leprosy cases are still appearing from all these countries. In this situation one has to be confident to diagnose leprosy. This review paper highlighted already explored antigens for diagnosis purposes and finally suggested better combinations of protein antigens of *M. leprae* versus immunoglobulin as detector antibody to be useful for leprosy diagnosis.

## 1. Introduction 

*Mycobacterium leprae *is noncultivable bacteria in artificial media, which is generally grown in cooler region of host especially human beings [1]. We believed that after infection* M. leprae* facilitates its environment for its survival in host. On entry cell wall, cell membrane and secreted proteins of* M. leprae* would be the first to interact with host immune cells; that is, these proteins can stimulate host immune system. In our opinion, potential peptide antigens which interact with defense cells may be soft target for development of diagnostic tools. Screening of IgG, IgA, and IgM response to antigens of* M. leprae* could shortlist the potential candidate antigens. Similar to other living beings, in a structural and functional unit, proteins are elaborate major portion of* M. leprae* cytosol and cell membrane, many of which are able to evoke antibody response in the host. WHO's global strategy for further reducing the leprosy burden and sustaining leprosy control activities, in all endemic communities, could not be fulfilled in absence of potential diagnostic tools. The accurate diagnosis of leprosy is the urgent need of all aspects of leprosy control. Overdiagnosis will lead to unnecessary treatment and sentimental stigma of persons. Underdiagnosis will be a way allowing for spread of disease. The ideal diagnostic test should be able to detect all leprosy patients (100% sensitivity) and indicate absence of* M. leprae *in healthy individuals (100% specificity). The sensitivity and specificity can be determined by comparison with true negative and true positive obtained in another reliable, well-established (gold standard) test. Case of leprosy slit skin smear and histopathological test are considered to be reliable but due to many technical problems reliability of these tests could be affected. Thus these are not perfect tests [2–5]. The PGL-1 fraction is part of the cell envelope of* M. leprae *and induces the production of the specific humoral response against PGL-1 detected in patient serum [6–8]. Immunohistological test to stain PGL-1 antigen showed higher specificity than routine histopathology [9]. Further confirmation is sought by additional studies. The PGL-1 antibody assay in combination of skin lesion was found to have up to 77% sensitivity and 93% specificity in MB patients from Brazil [10]. In Nepal PGL-1 indicated 84% sensitivity with very low specificity [11]. PGL-1 testing has been reported to be useful in MB relapse detection [12]. PGL-1 IgM test in study among household contacts has proved importance of consanguinity for the development of anti-PGL-1 IgM antibodies in most of the contacts with a family history of leprosy [13]. However, all household contacts did not show development of leprosy and a small group of patients remains who will be untreated. At present diagnosis of leprosy generally depends on dermatological sign alone and skin smear tests. The macules are the most apparent signs, but of low predictive value. Nevertheless, they are an early but nonspecific sign of leprosy and are often neglected by the patient or physicians. Other than macules, neurological (dysesthesia, motor disorders) signs may appear early on or be observed at a late stage in the progression of the disease [14]. Thus, newer serological tests based on protein antigen and or combination of protein antigen by combination of suitable IgM, IgG, or IgA may eventually overcome such difficulties.

## 2. Antigens Applied for Serodiagnosis

The antigenic analysis was hampered for* M. leprae,* as no one can be able to culture bacilli in artificial media for antigenic analysis. Investigators have used lepromatous nodules as a limited source of bacilli and identified unique* M. leprae *protein antigens that are not shared by other mycobacteria [15]. In 1968–1970 the armadillo (*Dasypus novemcinctus*) was identified as an animal model to study* M. leprae*. This animal was selected because of its long life span and lower body temperature (30–35°C) [16, 17]. Later on leprosy was reported in wild armadillos in the Southern United States, suggesting an association between natural leprosy disease in humans and armadillos. In 1985, experimentally infected armadillos serum and whole blood were examined by Truman to detect antibodies against the* M. leprae *major antigens using enzyme-linked immunosorbent assay (ELISA) for immunoglobulin M (IgM) antibodies to the species-specific phenolic glycolipid-I (PGL-1) antigen. However, these antibodies have no protective effect against* M. leprae *and are usually associated with high false-positive rates within leprosy endemic regions [18]. It is after discovery of armadillo as experimental animal [16] to culture* M. leprae*. Better knowledge of the specific antigens responsible for immune responses in leprosy patients is useful to develop a peptide or DNA vaccine against leprosy and to identify selective serological diagnostic reagents, since studies based on sodium dodecyl sulphate polyacrylamide gel electrophoresis [19–23] to 2-dimensional gel electrophoresis [24–35] to define the proteome of* M. leprae *have proposed the existence of specific antigens in* M. leprae* that are located in cell membrane, cell wall, and cytosolic for their utility in the serodiagnosis.

Heat stable antigens (12 kDa, 22 kDa, 28 kDa, 36 kDa, 41 kDa, and 86 kDa) were identified from* M. leprae* sonicates on using SDS-PAGE and treatment of gel with peroxidase-labelled anti-human IgG [36]. The lepromatous patients were more reactive against the defined antigens. Patient-wise variation in reactivity towards these antigens was found within this group. Similar variations were found by other authors measuring antibody reactivity against* M. leprae* antigens [37–40].

### 2.1. Whole* M. leprae* Sonicated Antigen

Whole* M. leprae* was used as an antigen [41] after removing cross-reactive component by absorbing the serum with cardiolipin, lecithin, BCG, and* M. vaccae* and employed in fluorescent leprosy antibody absorption (FLA-ABS) test. FLA-ABS test is being carried out in Japan, India, China, Korea, and many other countries of the Indian subcontinent. Most of the studies have showed 90% to 100% positivity in lepromatous and 70% to 80% in tuberculoid leprosy. Household healthy contacts of leprosy patients also showed 70% to 80% positivity indicating subclinical infection with* M. leprae* in the population.

### 2.2. 34 kDa Protein

Gene ML0158 has a product of 314 amino acids and (31374 da) of* Mycobacterium leprae* protein (http://www.sanger.ac.uk/Projects/M_leprae/CDS/ML0158.shtml). 34 kDa cell wall antigen is isologous to the immunodominant 34-kilodalton antigen of* M. paratuberculosis.* And similarly, 34 kDa isolog of* M. leprae* that also resides at the C terminus subcellular fractions of* M. leprae* provided unequivocal proof of the presence of two native versions of the 34 kDa protein. The antigen has been found to be lacking significant serological activity [42].

### 2.3. 35 kDa (MMP-1) Protein

It is a product of gene ML0841. Its 307 amino acid sequence has molecular weight of 33652 da. This protein can also be known as major membrane protein-I (http://www.sanger.ac.uk/Projects/M_leprae/CDS/ML0841.shtml). 35 kDa antigen of* M. leprae* was found in membrane fraction identified by Sinha et al. [43] and proved to be reactive to epitope on antibodies MAb ML04 in leprosy patient. This protein independently was identified by Hunter et al. [44] as a major membrane protein-I (MMP-I). It shows strong T-cell response in leprosy patients, elicits specific delayed type hypersensitivity, and stimulates IFN*γ* production also. This protein is absent in* M. bovis *and* M. tuberculosis*. It has detected the fact that 90% lepromatous cases and 40% tuberculoid patients have been reported as positive by using this antigen [43]. It also shows a weak positive response to tuberculosis patients. It has homologues in* M. intracellulare, M. avium, *and* M. paratuberculosis.*

### 2.4. ESAT-6 Protein

ESAT-6 in* M. leprae* found as homologue protein expressed that appearance in cell wall fraction shows only 36% homology in comparison to tuberculosis ESAT-6 [45, 46]. The anti-*M. leprae* ESAT-6 polyclonal and monoclonal antibodies and T-cell hybridomas reacted only with the homologous proteins and allowed B- and T-cell epitopes. The* M. leprae *ESAT-6 shows promise as a specific diagnostic agent for leprosy [46]. There is also a probable secreted antigen, product of gene ML0050 having molecular weight of 10964 Da and known as 10 kDa protein. This protein is a member of ESAT-6 protein and resembles culture filtrate protein-10 (CFP-10) of* Mycobacterium tuberculosis* (http://www.sanger.ac.uk/Projects/M_leprae/CDS/ML0050.shtml).

### 2.5. 10 kDa Protein

This is a product of gene groES ML0380. It has molecular weight of 10800 Da with known one hundred amino acids (http://www.sanger.ac.uk/Projects/M_leprae/CDS/ML0380.shtml). 10 kDa heat shock protein found in cell wall fraction is an important antigen recognized by T-cells, also known as chaperonin-10 (cpn-10). It responds to approximately 1/3rd of the* M. leprae* reactive T-cells in the patients with tuberculoid leprosy [47]. It elicits DTH response in* M. leprae* sensitized guinea pig. It lacks specificity as it shows 90% identity with its* Mycobacterium tuberculosis *counterpart. It has a flexible region, known to interact with cpn-60.

### 2.6. 15 kDa Protein

It is a product of gene lsr2 ML0234 and has molecular weight of 12165 Da. There are one hundred and twelve amino acids found in this antigen. Homologues are present for this antigen in* Streptomyces coelicolor *(http://www.sanger.ac.uk/Projects/M_leprae/CDS/ML0234.shtml). This 15 kDa non-fusion protein from cell wall have shown strong reactivity with LL patients to screen mycobacterial *λ*gt11 libraries. Antigen is also recognized by B-cell epitopes which recognize antibodies of patients from different geographical region. Antigen has property of clear recognition of human T-cells from leprosy patients [48, 49].

### 2.7. 18 kDa Protein

Gene hsp18 ML1795 has a product of protein of molecular weight of 16707 Da and a 148 amino acid sequence. The protein antigen is known as 18 kDa heat shock protein (http://www.sanger.ac.uk/Projects/M_leprae/CDS/ML1795.shtml). The* M. leprae *18 kDa provides 70% sensitivity among LL patients and about 72% among BT cases. It has been also found to be cross-reactive with sera from tuberculosis patients [50].

### 2.8. 21 kDa Protein

This conserved hypothetical protein has molecular weight of 24521 Da and contains about 228 amino acids. It is a product of gene ML2200 (http://www.sanger.ac.uk/Projects/M_leprae/CDS/ML2200.shtml). The* M. leprae* surface shows a marked protein (SDS predicted MW 28 kDa) for myelin producing Schwann cells; a surface-exposed laminin binding protein (LBP) of molecular mass 21 kDa (ML-LBP21) (found after peptide sequencing) may be an important virulence factor. Recombinant ML-LBP21 shows response against monoclonal antibodies [51, 52]. Rambukkana et al. [53] described how the G-domain of the laminin-*α*_2_ chain in the basal lamina that surrounds the Schwann cell axon unit serves as an initial neural target for* M. leprae*. By using human-*α*_2_ laminin as probe, a major 28 kDa protein in the* M. leprae *cell wall fraction was identified. The 28 kDa protein functions as critical surface adhesive that facilitates the entry of* M. leprae* in Schwann cells. Pessolani and Brennan [29] had also described a similar 28 kDa protein as a key bacterial ligand in* M. leprae *Schwann cells interaction and have shown that it is a member of histone like protein family.

### 2.9. 30 kDa Protein

This is also a conserved hypothetical protein. It is product of gene ML0849 having 283 amino acids. The molecular weight of this protein is about 30520 da (http://www.sanger.ac.uk/Projects/M_leprae/CDS/ML0849.shtml). The* M. leprae* 30/31 kDa protein is only known as a secreted protein that induces strong humoral and cellular immune response and it contains at least two fibronectin binding sites. This 30/31 kDa protein not only is important in the immune response against* M. leprae* but may also have a biological role in the interaction of this bacillus with the human host [54].

### 2.10. 45 kDa Protein

This is a product of gene ML0411 having molecular weight of about 42467 da. And it contains approximate four hundred and eight known amino acids (http://www.sanger.ac.uk/Projects/M_leprae/CDS/ML0411.shtml). The 45 kDa protein (antigen) found in the* M. leprae *sonicate shows that human T-cell response reflects infection with or exposure to* M. leprae*. It is a serine rich antigen found to give peripheral blood mononuclear cell proliferation response about 92.8% in tuberculoid leprosy cases, in lepromatous leprosy cases it was 60.6%, in leprosy contacts it was 88%, and in controls it was 10% [55]. Parkash et al. [56] have evaluated this antigen for MB and PB cases and found this serine rich molecule as highly specific for leprosy. There were about 94.4% MB and 36.8% PB found to be positive on using molecule as diagnostic tool.

### 2.11. MMP-II (Bfr) Protein

This antigen corresponds to gene* bfrA* ML2038 or pseudogene* bfrB* ML0075 (http://www.sanger.ac.uk/Projects/M_leprae/CDS/ML0075.shtml) and there are one hundred and fifty-nine amino acids known for bfrA ML2038 with a molecular weight of 18263 da (http://www.sanger.ac.uk/Projects/M_leprae/CDS/ML2038.shtml). Bacterioferritin (Bfr) is a major membrane protein II (MMP-II) observed abundantly in* in vivo *grown* M. leprae *involved in acquisition and storage of iron. In the context of Johne's disease,* M. paratuberculosis *Bfr is an immunodominant B-cell antigen, and it is the key component of a diagnostic test [57]. Therefore relevance of* M. leprae *Bfr as an antigen for the purpose of diagnosis can be explored.

### 2.12. TR/Trx Protein

This protein is a gene product of gene trxB ML2703 and its molecular weight is 49047 da. The protein has 458 amino acids (http://www.sanger.ac.uk/Projects/M_leprae/CDS/ML2703.shtml). This bifunctional hybrid protein thioredoxin/thioredoxin reductase (TR/Trx) found only in* M. leprae *among* Mycobacteria *as TR and TRX are separate proteins in other* Mycobacteria*. The N terminus of it is homologous to the TR and C terminus to Trx. This protein is active by itself and its activity involves intramolecular interaction between TR and Trx domains. This protein also shows the intramolecular interaction in excess of TR or Trx [58]. The diagnostic role of the antigen seems to be absent as other bacteria have protein but only in form of two separate molecules.

### 2.13. 65 kDa Protein

There is a 65 kDa protein found in both membrane and cytosol of* M. leprae *[59]. It is* Chepronin* 65 kDa GroEL-2 related to the family of heat shock proteins which is the major protein present in host derived* M. leprae* [44]. There are only 29% leprosy patients responding to it.

### 2.14. Ahpc Protein

Ahpc protein of* M. leprae* shows similarity with the C22 unit of alkyl hydroperoxide reductase (Ahpc) from* Salmonella typhimurium*, a detoxifying enzyme that reduces organic hydroperoxides to their corresponding alcohols. Homologous* M. leprae *AhpC protein is a member of the AhpC-thiol specific family of enzymes with antioxidant activities. This protein plays a key role in the survival of* M. leprae* in the midst of high concentration of oxygen-reactive species produced by macrophages [29].

### 2.15. CysA Protein

There is a protein for cysteine biosynthesis and sulfur assimilation in* M. leprae *that is known as CysA protein. The gene for this also encodes a putative sulfate sulfurtransferase enzyme. But it shows high similarity to* Saccharopolyspora erythraea *CysA and both of them show homology to human liver protein rhodanese [29].

## 3. Recombinant Proteins in Diagnosis of Leprosy

Based on* M. leprae* cDNA library screening results [21] thirty-three protein antigens were ML0022, ML0051, ML0098, ML0176, ML0276, ML0393, ML0405, ML0489, ML0491, ML0540, ML0810, ML0811, ML0840, ML1383, ML1556, ML1632, ML1181, ML1481, ML1633, ML1685, ML2028, ML2044, ML2055, ML2203, ML2331, ML2346, ML2358, ML2380, ML2541, ML2603, ML2629, ML2655, and ML2659; recombinant proteins were studied for immune response [60]. Of these, ML0405, ML2055, and ML2331 were incubated with blood from TT/BT and healthy household contacts (of LL/BL patients) groups; the proteins induced strong IFN*γ* production but weak or absent antibody responses, although ML0405 and ML2331 proteins were well recognized by serum IgG from LL/BL leprosy group [60]. Researchers suggested that the antibody response to* M. leprae* recombinant proteins was dependent upon their ability to induce cellular responses and indicates that only a limited number of* M. leprae* antigens contained T-cell and B-cell epitopes that are immune reactive in the context of disease (ML0405, ML2055, and ML2331). Sampio et al. [60] suggested a combination of whole blood assay prior to serological assays for beneficial protein screening. Most of these antigens neither induced IFN*γ* secretion nor showed IgG reactivity. The antigens showing IgG reactivity can be a potential combination with PGL-1 antigen for leprosy diagnosis.

## 4. Peptide Based Serodiagnosis 

It is hypothesised that during dormancy of disease most patients are subclinically infected and this subclinical infection could be source of* M. leprae* transmission. The modern tools and improved bioinformatics to study genome sequence of* M. leprae* have opened new door of possibilities for leprosy research. Now we are positioned to predict more relevant* M. leprae* proteins and potential human leukocyte antigen (HLA) class I and class II epitopes that can activate T-cells [61]. These postgenomic approaches have proposed novel* M. leprae* protein or peptide-specific T-cell responses to identify* M. leprae*-exposed or* M. leprae*-infected individuals [19, 20, 26, 62–64].

Antigenic proteins typically contain multiple peptide epitopes. Comparably, antigenic proteins have better diagnostic potential due to reduced or absent T-cell cross-reactivity [26] and [65]. Analysis of* M. leprae* peptides or pools of peptides in geographically different endemic regions could provide unique 138,938 20-mer peptide sequences derived from 1,546 different* M. leprae* candidate proteins. To reduce the number of candidate peptides for BLAST, Bobosha et al. [66] proposed selected peptides derived from genes in the functional classification group IV.A (virulence) (including the following 13 genes: ML0360, ML0361, ML0362, ML0885, ML1214, ML1358, ML1811, ML1812, ML2055, ML2208, ML2466, ML2589, and ML2711) (http://www.sanger.ac.uk/Projects/M_leprae/Ml_gene_list_hierarchical.shtml, currently designated as “genes involved in virulence, detoxification and adaptation” or “genes involved in cell wall and cell processes” on http://mycobrowser.epfl.ch/leprosy.html). These peptides induced T-cell reactivity in leprosy patients or healthy individuals living in regions. The potential diagnostic reagents were predominantly derived from ML1601, ML2055, ML1358, and ML1214. There was region-wise variation in response to these peptides. The discrepancy among peptide's responses could be due to variation in HLA polymorphism; however, peptides have potential use in estimating the level of* M. leprae* exposure [66]. ML2055 has also been reported to induce strong serological responses in lepromatous patients [60]. The immune response against* M. leprae* infection is a collective/synergistic response of various immune cascades that involve the induction of both cytokines and chemokines by innate and adaptive immune cells [66]. The main advantage arising from the use of synthetic peptides compared with the use of proteins is that peptides less frequently induce T-cell cross-reactivity [20] However, because of the HLA-restriction of peptides that are recognised by T-cells, single peptides are not able to cover diverse populations.

## 5. Recent In-House Studies towards Leprosy Diagnosis

Most of these protein antigens have been reported to induce T-cell response from tuberculoid leprosy patients and their contacts* in vitro.* However, majority of these antigens have been shown to be cross-reactive with homologues identified in other mycobacterial species. Hence, such antigens would not be useful as diagnostic reagents.

**Figure 1:**
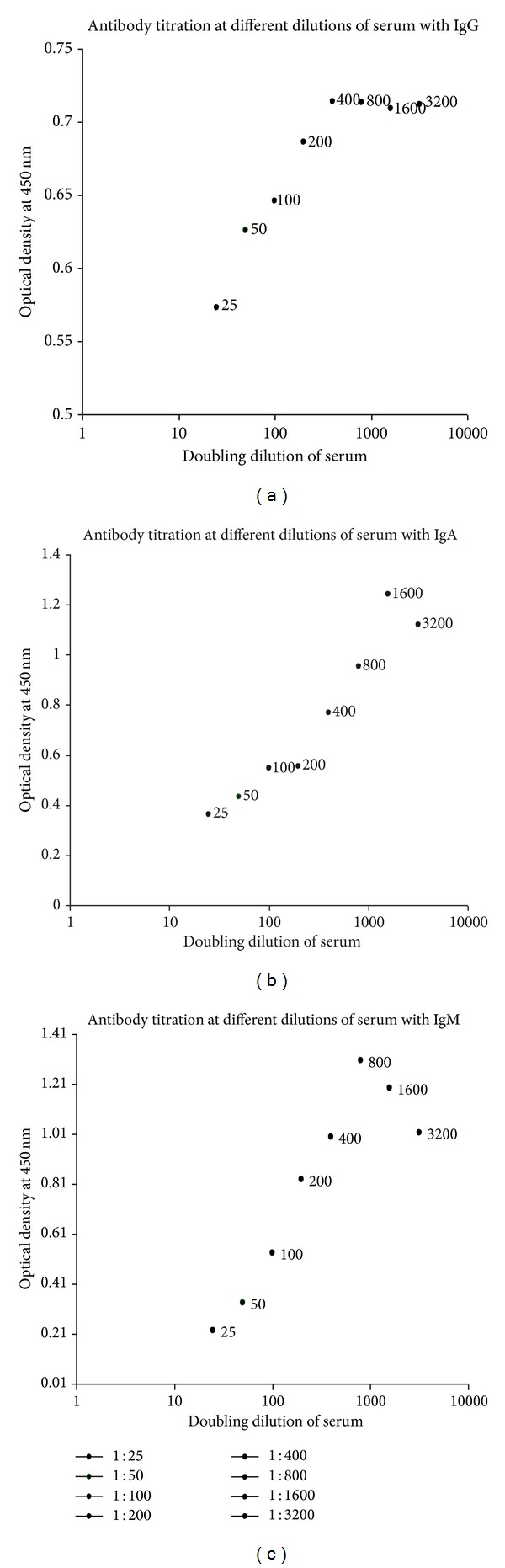
Bacterial index (BI) and MLF positive serum samples were used for calculating the best dilution of serum to recognize samples having anti-*M. leprae* antibodies. Results indicated that* M. leprae *infected human serum was reacted with the whole* M. leprae* sonicated antigen proteins where detector antibody was (a) IgG, (b) IgA, or (c) IgM. Examination of sera gives a general pattern found as above. Thus selected dilution of serum was 1 : 400 1 : 800 and 1 : 1600 for immunoblots with IgG, IgM, and IgA, respectively.

In the past decade, clinically defined leprosy patients were analyzed by us for BI indices, MLF test, and indirect ELISA. Sera from healthy individuals working in various laboratories of the institute were tested for defining cut-off value. Whole* M. leprae* sonicate antigen was recognized by leprosy patient sera (Figure 1); equal or more than a cut-off (calculated by mean optical density + 2 standard deviation for healthy controls) was considered to have acceptable antibodies titer (Figure 1). Sera from selected LL with BI positive (MB) and BT with BI negative (PB) leprosy were tested in triplicate, and the mean absorbance for control wells without antigen was subtracted from that for sample wells before analysis. Two-dimensional gel electrophoresis (2DE) separated proteins of* M. leprae* (cytosol, cell wall, and cell membrane) were immunoblotted with anti-human IgA/IgM/IgG to obtain immunoblots against sera of leprosy patients, tuberculosis patients, and healthy individuals. The observed immunogenic antigen of* M. leprae* present specifically only in leprosy sera would then be recognized and analysed statistically.

### 5.1. Estimation of Antibody Titers, in Sera Samples, against Whole* M. leprae* Protein Antigens

The* M. leprae *flow(MLF) test and bacterial index (BI) (slit skin smear) of patients were used to select samples to calculate higher antibody titer to identify potential protein antigens that could ultimately serve as the basis for an immunodiagnostic test for leprosy. To check the potential specificity of MLSA, MLMA, and MLCwA, we selected serum samples with appropriate antibody titer which was given by indirect ELISA. Initially, sera samples were collected and classified as LL/BL and BT [67]. However, since they were clinically leprosy patients, serological analysis was performed with the MLF test and indirect ELISA which gave us a set of serum samples of leprosy patients that were likely to be strictly having higher load of antibodies against* M. leprae*.

### 5.2. Selection of Serum Samples Having Applicable Anti-*M. leprae* Antibody Titer

Leprosy patients were classified as multibacillary (MB) and paucibacillary (PB) on the basis of clinical criteria given in guidelines of WHO and NLEP [68]. Briefly, patients with more than 5 lesions and/or 2 or more affected nerve trunks were classified as MB (23LL/2BL/4BB/41BT/14N). Patients with up to 5 lesions with or without nerve thickenings (<2) were included in PB group (13BT/1I/3N). All selected LL patients had bacilli in the skin smear and were positive with MLF whereas BT patients were negative for both. 92.0% of the MB patients and 32.0% of PB patients were serologically positive by the ML Flow test [69]. In- house calculated cut-off values regarding IgA, IgM, and IgG were 0.206, 0.325, and 0.5, respectively.

### 5.3. Analysis of ELISA and Immunoblot Observations

**Figure 2:**
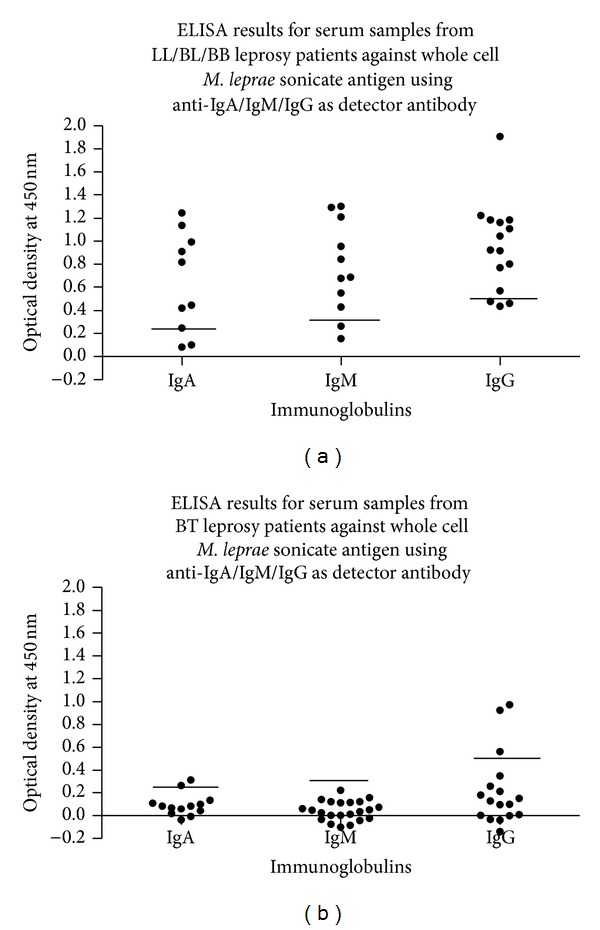
Leprosy patients responses to IgG, IgA, and IgM above cut-off values were selected as positives. Here anti-IgA/IgM/IgG was taken as detector antibody for (a) LL/BL/BB and (b) BT leprosy patient.

Indirect ELISA for calculating antibodies level for whole* M. leprae* sonicated antigen (WMLS) in serum samples where we have found serological positivity of 10/22 (45.45%), 9/35 (25.71%), and 15/32 (46.88%) in leprosy cases with anti-IgA, IgG, and IgM was taken as detector antibody in leprosy patients, respectively. Indirect ELISA for calculating antibodies level for whole* M. leprae* sonicated antigen (WMLS) in serum samples where OD values have cut-off values (0.206, 0.5, and 0.325) (Figure 2) at dilution 1 : 1600, 1 : 400, and 1 : 800 of serum, respectively, with anti-IgA, IgG, and IgM was taken as detector antibody in untreated leprosy patients, respectively. Immunoblot of cytosolic* M. leprae* fractions had majority of antigens (18/39 spots, Table 1). Of these, major numbers 11/18 spots were paired with IgG antibodies in sera (4 with anti-IgA and 3 with anti-IgM). On further analysis, majority of antigens from cytosol and cell wall raised IgG antibody titer while cell membrane has major number of antigens to raise IgA antibodies. Most of antigens of* M. leprae* responsible to raise IgM titer were also located in cell membrane. The samples were collected from patients and healthy individuals who are resident in India where people are supposed to be immunized at the age of 6 weeks with BCG. That is why very few of these antigen spots were found as specific to leprosy in study. Hence, immunoblot based leprosy specific antigens were identified by using MALDI-TOF/TOF.

Our assays (based on IgG, IgA, and IgM) indicated that lepromatous leprosy patients have higher antibody titer in comparison to BT patients. Therefore the study was focused towards investigating the best immunoglobulin and antigen combination to diagnose higher number of BT patients. All proteins of* M. leprae* would not react in similar fashion in all individuals and they rely on the patient's immunity. On considering immunoglobulins, IgG responded against more numbers of antigens (18) than IgA (11) or IgM (10). Among these, MALDI TOF/TOF based leprosy specific antigenic repertoires of only 8 proteins were found to belong to membrane fraction [70]. Reported antigen MAL5 was recognized with IgA having 82.6% sensitivity with maintaining specificity up to 54.5% which is the best among all these specific antigens. MALDI-TOF-MS/MS of MAL5 indicated it as MMP-I. After considering immunoblot and MALDI results MAL5 is an isoform of MMPI [70]. Immunoblot based leprosy specific antigens (in comparison to* Mycobacterium tuberculosis*) were not supposed to be potential diagnostic reagent if they were not specific to* M. leprae* in MALDI-TOF/TOF observations (found homologous with other actinomycetes) (unpublished data).

Therefore study suggested that if one has chosen cell membrane protein for leprosy diagnosis, then IgA will be a better detector antibody. Further searching peptide in these antigens can provide better diagnostic tool when IgA will be a detector antibody. The* M. leprae* had wide number of antigens which were cross-reactive to other bacteria. Among 39 investigated antigens for diagnosis purpose, antigen MAL5, recognized with IgA, having 82.6% sensitivity with maintaining specificity up to 54.5%, is the best among all above mentioned antigens. This study has also opened a door of hope to search a protein (peptide) to develop vaccine to prevent leprosy.

## 6. Future of Leprosy Serodiagnosis

Hungria et al. [71] investigated serologic reactivity to the novel* M. leprae *proteins 46f and 92f,“leprosyIDRI diagnostic-1” (LID-1), and ML0405 and ML1213 using IgG as detection antibody and suggested that enrichment of PGL-1 + IgM test with any of these antigens + IgG could improve serodiagnosis of leprosy cases.

Similarly, peptides from these and above-mentioned proteins can provide specific immune responses in leprosy patients in an endemic region. Thus synergistic combination of proteins/protein, protein-PGL-1, or peptide could be useful to develop a rapid diagnostic test for the early detection of* M. leprae* infection and epidemiological surveys of the incidence of leprosy, of which little is known. Still in all these sets one has to know the most suitable detector antibody. Studies have suggested that IgG makes combination with larger number of* M. leprae* proteins for diagnosis but so far tested antigens could not be able to provide high specificity. Considering above-mentioned antigens and in-house study,* M. leprae* had higher number of specific antigens in cell membrane if visualized in immunoblots using IgA and IgM as detector antibodies.

## 7. Conclusion

Immunoblot and ELISA based study of* M. leprae* antigens can suggest highly specific and sensitive protein molecules from endemic region of leprosy that can be used as diagnostic reagent if proper immunoglobulin was a detector antibody. We can also develop a peptide based on synergistic combination for serodiagnostic test to identify presence of* M. leprae *in the patient's body.

## Figures and Tables

**Table 1:** The number of proteins (antigens) reactive to various sera samples of leprosy patients which were not presented by healthy people's and tuberculosis patients' sera.

Groups	IgG	IgA	IgM	Total
Cytosolic proteins (MLSA)	11	04	03	18
Cell wall proteins (MLCwA)	04	01	02	07
Cell membrane proteins (MLMA)	03	06	05	14

Total leprosy patients' sera reactive spots	18	11	10	39
